# Perinatal Hypoxia-Ischemia Reduces ****α****7 Nicotinic Receptor Expression and Selective ****α****7 Nicotinic Receptor Stimulation Suppresses Inflammation and Promotes Microglial Mox Phenotype

**DOI:** 10.1155/2014/718769

**Published:** 2014-03-17

**Authors:** Sansan Hua, C. Joakim Ek, Carina Mallard, Maria E. Johansson

**Affiliations:** Department of Physiology, Institute of Neuroscience and Physiology, The Sahlgrenska Academy, University of Gothenburg, P.O. Box 432, 405 30 Gothenburg, Sweden

## Abstract

Inflammation plays a central role in neonatal brain injury. During brain inflammation the resident macrophages of the brain, the microglia cells, are rapidly activated. In the periphery, **α**7 nicotinic acetylcholine receptors (**α**7R) present on macrophages can regulate inflammation by suppressing cytokine release. In the current study we investigated **α**7R expression in neonatal mice after hypoxia-ischemia (HI). We further examined possible anti-inflammatory role of **α**7R stimulation *in vitro* and microglia polarization after **α**7R agonist treatment. Real-time PCR analysis showed a 33% reduction in **α**7R expression 72 h after HI. Stimulation of primary microglial cells with LPS in combination with increasing doses of the selective **α**7R agonist AR-R 17779 significantly attenuated TNF**α** release and increased **α**7R transcript in microglial cells. Gene expression of M1 markers CD86 and iNOS, as well as M2 marker CD206 was not influenced by LPS and/or **α**7R agonist treatment. Further, Mox markers heme oxygenase (Hmox1) and sulforedoxin-1 (Srx1) were significantly increased, suggesting a polarization towards the Mox phenotype after **α**7R stimulation. Thus, our data suggest a role for the **α**7R also in the neonatal brain and support the anti-inflammatory role of **α**7R in microglia, suggesting that **α**7R stimulation could enhance the polarization towards a reparative Mox phenotype.

## 1. Introduction

Perinatal hypoxia-ischemia (HI) is a major cause of brain injury in newborns, resulting in an increased risk of developmental impairment and permanent neurological deficits such as cerebral palsy and mental retardation [1]. Inflammation plays a central role in the development of brain injury in newborns [2]. Both neonatal hypoxia-ischemia and stroke trigger an inflammatory response [3, 4] and experimental studies show that inhibition of proinflammatory mediators is neuroprotective [5, 6].

Microglia, the resident macrophage of the brain, are central in this process being the main cell providing immunosurveillance in the brain. During pathological conditions, such as hypoxia-ischemia, microglia are rapidly activated with antigen presentation and secretion of cytokines and other inflammatory mediators as a consequence [7]. In the periphery, macrophages are highly dynamic cells that can be polarized into different macrophage phenotypes depending on the microenvironment, that is, the classical proinflammatory M1 macrophage and the wound-healing M2 macrophage being the most discussed phenotypes [8, 9]. Recently, also other macrophage phenotypes have been described, the Mox macrophage that develops in response to oxidative stress [10] and the M4 macrophage, induced by the platelet-derived cytokine CXCL4 [11]. Interestingly, also primary microglial cells can be polarized* in vitro* into different microglial phenotypes [12].

In the brain, nicotinic acetylcholine receptors (nAChRs) contribute to regulation of neuronal plasticity [13] and neuroprotection [14, 15]. These ion channels, forming homo- or heteropentamers, have been suggested to play important roles in neurodegenerative diseases such as Alzheimer's disease [16], Parkinson's disease [17], and schizophrenia [18, 19]. The most abundant nicotinic receptors in the brain are the *α*4*β*2 (*α*4*β*2R) and the *α*7 receptors (*α*7R). In the periphery, the *α*7 nicotinic receptor (*α*7R) can modulate inflammation [20], that is, signaling via the *α*7R inhibits cytokine release, thereby suppressing inflammation and providing protection against tissue damage in inflammatory states [21]. The *α*7R is expressed by leucocytes; however, macrophages have been identified as the primary effector cell [22].

Recently, stimulation of the *α*7R was described to have a neuroprotective role in adult brain injury [23], and to be expressed by microglial cells [24, 25], however, little is known about the *α*7R expression in the neonate and its role in perinatal brain injury. In the present study we hypothesized that the expression of *α*7R is decreased after perinatal hypoxia-ischemic brain injury and that stimulation of *α*7R with a selective *α*7R agonist, AR-R 17779, has an anti-inflammatory effect on microglia. Further, we investigated the microglial phenotype after stimulation with *α*7R agonist.

## 2. Material and Methods

### 2.1. Animals

C57BL/6J mice obtained from Charles River were housed and bred in a room with 12 h light/dark cycle. Water and standard laboratory food were available* ad libitum*. All procedures involving animals were approved by the regional ethics committee of Gothenburg and performed according to the Swedish guidelines for the Care and Use of Laboratory animals.

### 2.2. Hypoxia-Ischemia Model

Hypoxia-ischemia (HI) in neonatal mice was performed as previously described [26]. In brief, at postnatal day (P) 9-10, the left common carotid artery was ligated under isoflurane anesthesia. After ligation, the wound was closed, anesthesia discontinued, and the mice were allowed to recover for one hour. After recovery, the mice were placed in an incubator circulated with firstly normal air for 10 minutes, secondly with a humidified gas mixture (10.00 ± 0.01% oxygen in nitrogen) for 45 minutes, and thirdly with normal air again for 10 minutes. Incubator temperature was kept at 36°C throughout the experiment. Thereafter, the pups were returned to their dam until sacrifice. The method induces hypoxic-ischemic injury to the left cerebral hemisphere [26]. At 24 and 72 h after HI the animals were terminally anesthetized and intracardially perfused with saline to remove blood from the brain. Brains were collected, snap frozen in N (*l*) and stored at −80°C until further analysis. Control mice underwent sham surgery. Mice at P9-10 were used given that their brains are approximately at a developmental stage equivalent to the near-term human infant [27].

### 2.3. Microglial Cell Culture

Mixed glial cell cultures were prepared from whole brains of P2-3 mice. Brains were homogenized by pipetting in Dulbecco's modified Eagle's medium (DMEM) with 20% fetal bovine serum (FBS) and 1% Penicillin-Streptomycin (Sigma Aldrich, Stockholm, Sweden) followed by filtration through 70 *μ*m cell strainer (BD Biosciences, Stockholm, Sweden). Cells were seeded in DMEM 20% FBS and 1% Penicillin-Streptomycin in 75 cm^2^ flasks (Sarstedt AB, Helsingborg, Sweden) and cultured in 5% CO_2_/95% air at 37°C. After 7 days* in vitro* the medium was replaced with DMEM with 10% FBS/1% Penicillin-Streptomycin. Mixed glial cells reached confluency after 14 days* in vitro*. Primary microglia were mechanically isolated by using a reciprocating shaker at 250 rpm for 3 hours at 36°C. Microglia cells were pelleted via centrifugation at 250 g for 10 min, resuspended in DMEM with 2% FBS/1% Penicillin-Streptomycin and 200 000–250 000 cells were plated/well in 12-well plates (BD Biosciences, Stockholm, Sweden). Following incubation for 24 h, cells were stimulated with LPS (10 ng/mL in PBS, List Biological Laboratories Inc., Campbell, CA) with or without the *α*7R agonist AR-R 17779 (Tocris Bioscience, Bristol, UK) with the indicated doses. AR-R 17779 was dissolved in Dimethyl Sulfoxide (DMSO) and then diluted in culture media to a maximal final concentration of 3.3% DMSO. After 4 h incubation, supernatant and cells were harvested and stored at −80°C for further analysis.

### 2.4. RNA Extraction, cDNA Synthesis, and Gene Expression Analysis

Brains collected after HI and microglia samples obtained from cell cultures were homogenized by pipetting in RNase free PBS and using 30G insulin syringes (BD Biosciences, Stockholm Sweden) with RLT buffer (Oiagen GmbH, Hilden, Germany), respectively. RNA was extracted by using the RNAeasy Lipid Tissue Mini/Micro Kit (Qiagen GmbH, Hilden, Germany) according to the manufacturer's protocol. RNA concentration was determined with NanoDrop analysis (NanoDrop Products, DE, USA). QuantiTect Reverse Transcription kit (Qiagen GmbH, Hilden, Germany) was used to synthesize first strand cDNA according to the manufacturer's protocol.

Real-time PCR analysis was run on a LightCycler 480 (Roche Diagnostics GmbH, Mannheim, Germany) using the following cycling program: denaturation at 95°C for 10 minutes followed by 45 cycles of denaturation at 95°C for 15 seconds and annealing/extension at 60°C for 4 seconds and 72°C for 8–12 seconds, respectively. Melting-curve analysis was performed to ensure that only one PCR product was obtained. PCR products were further validated on agarose gel. All samples were run in duplicate. Intersample differences were limited to 0.5 cycles and samples with >0.5 cycles difference being excluded from the analysis. The following primers were used: *α*7R (chrna7, QT00143626), *α*4R (chrna4, QT00144662), *β*2R (chrnb2, QT00127708), CD86 (QT01055250), iNOS (QT01547980), CD206 (mrc1, QT00103012), Arginase 1 (Arg1, QT00134288), heme oxygenase (Hmox1, QT00159915), and sulforedoxin-1 (Srx1/npn3, QT00289443, all from Qiagen). The expression level of each target gene was normalized against the reference gene YWHAZ (tyrosine 3-monooxygenase/tryptophan 5-monooxygenase activation protein, QT00105350), calculated as 2^−ΔΔCT^, where ΔCT was the CT of the target gene after subtracting the CT value of the reference gene and ΔΔCT was the CT value corrected by the average CT of each group.

### 2.5. TNF*α* and IL-6 Analysis in Cell Culture Supernatants

Microglial cell supernatants were obtained by collecting the media of stimulated cells followed by centrifugation at 8000 g for 3 min. The supernatants were transferred to new tubes and analysis of TNF*α* and IL-6 levels were performed by ELISA (BioLegend Inc., San Diego, USA) according to the manufacturer's protocol.

### 2.6. Statistical Analysis

All data are presented as mean ± SEM. Normality was tested using the Shapiro-Wilk normality test and parametric or nonparametric tests were used accordingly. Normally distributed data were analyzed with ANOVA followed by Dunnett's or Tukey's multiple comparison test. Data that did not fulfill the test for normality was analyzed by Kruskall-Wallis one-way analysis of variance followed by Dunn's multiple comparison test. All statistical analyses were performed by SPSS (IBM SPSS Statistics 20, IBM Corporation, CHI, USA) or Prism (GraphPad Prism 5, GraphPad Software, Inc., CA, USA). The significance level was set to *P* ≤ 0.05.

## 3. Results and Discussion

### 3.1. HI Decrease *α*7R Expression at 72 Hours in the Neonatal Brain

**Figure 1:**
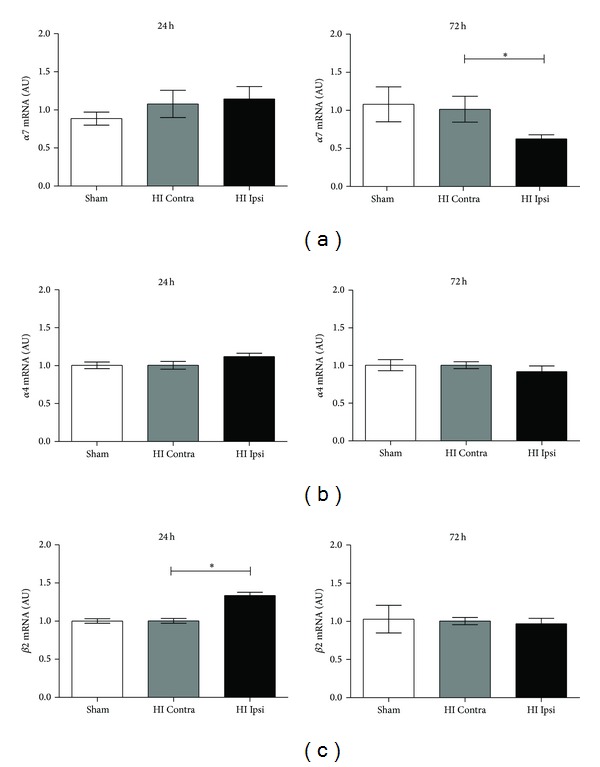
Decreased *α*7R expression 72 h after HI. Gene expression analysis of *α*7R, *α*4, and *β*2 receptor subunit levels in brains 24 and 72 h after hypoxia-ischemia (HI) in neonatal mice at age P9-10 ((a)–(c)). (a) At 24 h after HI there was no difference in gene expression of *α*7R; however, at 72 h *α*7R gene expression was significantly reduced in the injured (ipsilateral, Ipsi) hemisphere compared to the noninjured (contralateral, Contra) hemisphere. (b) Gene expression of *α*4R subunit was not influenced at 24 or at 72 h after HI. (c) *β*2R subunit expression was significantly increased in the injured (Ipsi) hemisphere compared to the noninjured (Contra) hemisphere at 24 h after HI. There was no difference in gene expression of *β*2R subunit at 72 h after HI. There was no difference between the sham animals (ipsilateral hemisphere) compared to the noninjured contralateral hemisphere in HI mice. Gene expression was normalized to YWHAZ and analyzed using ΔΔCT method. Data are expressed as mean ± SEM, *P < 0.05 for contralateral versus ipsilateral hemisphere in HI mice, *n* = 4-5 mice/group.

The expression of nicotine receptors are decreased in patients with neurodegenerative disorders such as schizophrenia [28] and Alzheimer's disease [16]. Also the expression of *α*7R is decreased in experimental models of adult brain injury [29]. Little is known of the expression of *α*7R in the neonatal brain or following brain injury in neonates. Hence, we investigated the expression levels of the *α*7R in a well-documented HI model in neonatal mice. Mice at P9-10 were chosen, as this can be approximated to a term human infant in terms of brain development [27]. At 24 h after HI there was no difference in *α*7R expression (Figure 1(a)); however, at 72 h after HI *α*7R gene expression was decreased by 33% in the injured versus noninjured hemisphere (Figure 1(a)). The gene expression of *α*7R was not altered in the noninjured hemisphere compared to mice undergoing sham surgery (Figure 1(a)). Thus, similar to traumatic brain injury in adult [30], the *α*7R gene expression is diminished after neonatal brain injury.

In the brain, the most abundant nicotinic receptors are the *α*7R and the receptor consisting of *α*4 and *β*2 subunits, respectively (*α*4*β*2R) [31]. To explore whether HI influence a general change in nicotinic receptor expression or if this was specific for *α*7R we analyzed the expression of the receptor subunits of the *α*4*β*2R. The gene expression of the *α*4 subunit was not altered by HI (Figure 1(b)); however, *β*2 gene expression was significantly increased 24 h after HI (Figure 1(c)). This is particularly interesting since a recent study suggests that lack of, or, antagonists to *β*2-containing nicotinic receptors decrease brain injury in adult stroke [32]. Whether the increased *β*2 gene expression after HI contributes to brain injury in neonatal mice remains to be explored.

### 3.2. *α*7 Receptor Agonist Increase *α*7R Gene Expression in Microglial Cultures

**Figure 2:**
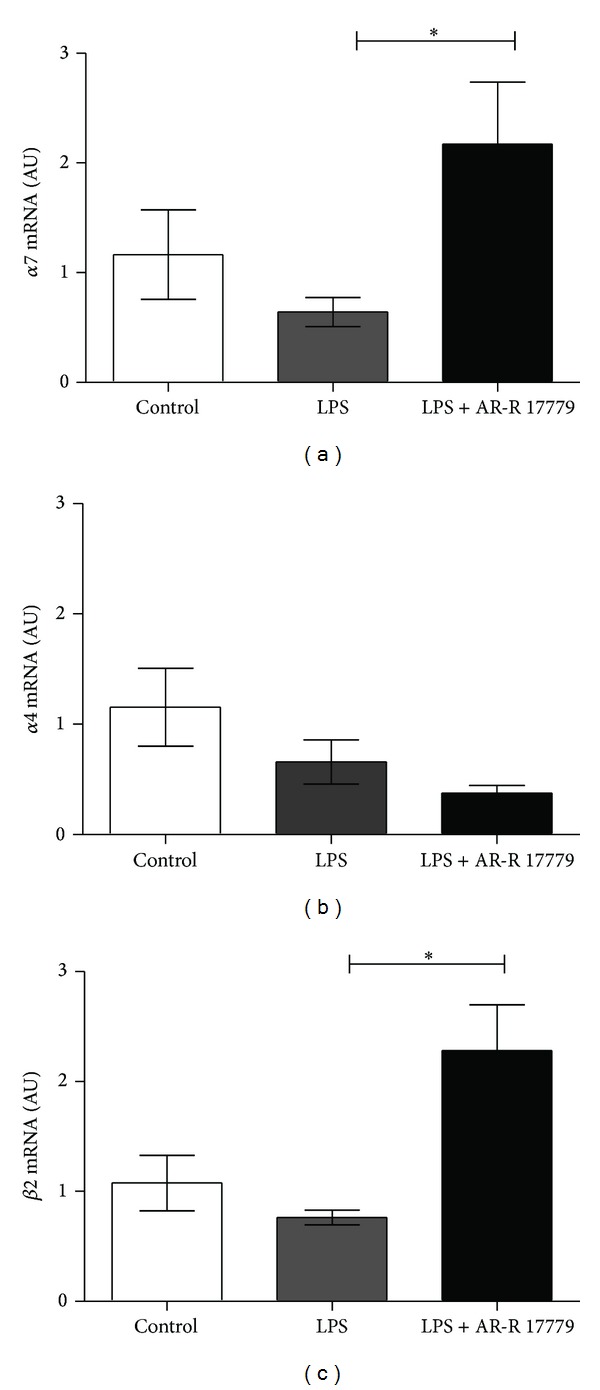
*α*7R agonist increase *α*7R gene expression in microglia cultures. Primary microglia cultures were stimulated with LPS (10 ng/mL) with or without *α*7R agonist AR-R 17779 (10 µM) for 4 h. Cells were collected and gene expression of (a) *α*7, (b) *α*4, and (c) *β*2 receptor subunits was investigated using real-time PCR. *α*7R agonist AR-R 17779 significantly increased *α*7R expression (a), whereas the *α*4 receptor subunit was not influenced by the treatment (b). Further, gene expression of *β*2 receptor subunits was increased by *α*7R agonist treatment (c). Graph represents pooled data from 4 independent experiments and *n* = 4/group. Gene expression was normalized to YWHAZ and analyzed using ΔΔCT method. Data are expressed as mean ± SEM, *P < 0.05.

After establishing that *α*7R is regulated in HI we sought to investigate its expression in microglial cultures and its possible anti-inflammatory effect. Primary rat and mouse microglial cultures express *α*7R [24, 25]. We could confirm these findings in the present study. We further explored the expression of *α*7R after proinflammatory stimulation, using LPS, with or without the selective *α*7R agonist AR-R 17779. Interestingly, *α*7R expression was not altered by LPS stimulation* per se*; however, *α*7R expression increased by 86% in microglial cells treated with LPS and *α*7R agonist AR-R 17779 (Figure 2(a)). Similar to the expression in the brain tissue, gene expression of the *α*4 subunit was not altered by the different treatments (Figure 2(b)). However, *β*2 gene expression was significantly increased by agonist treatment (Figure 2(c)). From our results we cannot determine whether this increase have functional implications [33].

Long-term treatment with nicotine, selective *α*7R agonists [34, 35], as well as, treatment with acetylcholine esterase inhibitors (AChE) [36], the enzyme responsible for acetylcholine degradation, increase *α*7R on protein level. Several mechanisms are suggested to be involved in agonist-induced upregulation of nicotinic receptors, for example, increased receptor trafficking to the surface, decreased cell surface turnover, increased subunit maturation, and decreased subunit degradation [35]. When comparing *α*7R expression between different mouse strains, *α*7R gene expression does not correlate with protein levels [37]. However, the mice in the previous study were not treated with *α*7R agonists, it is possible that *α*7R agonists treatment could influence *α*7R expression on both gene and protein level. Thus, further studies are needed to explore whether the increase in microglial *α*7R gene expression after agonist treatment is translated into protein.

### 3.3. *α*7 Receptor Agonist AR-R 17779 Decrease TNF*α* in a Dose-Dependent Manner

**Figure 3:**
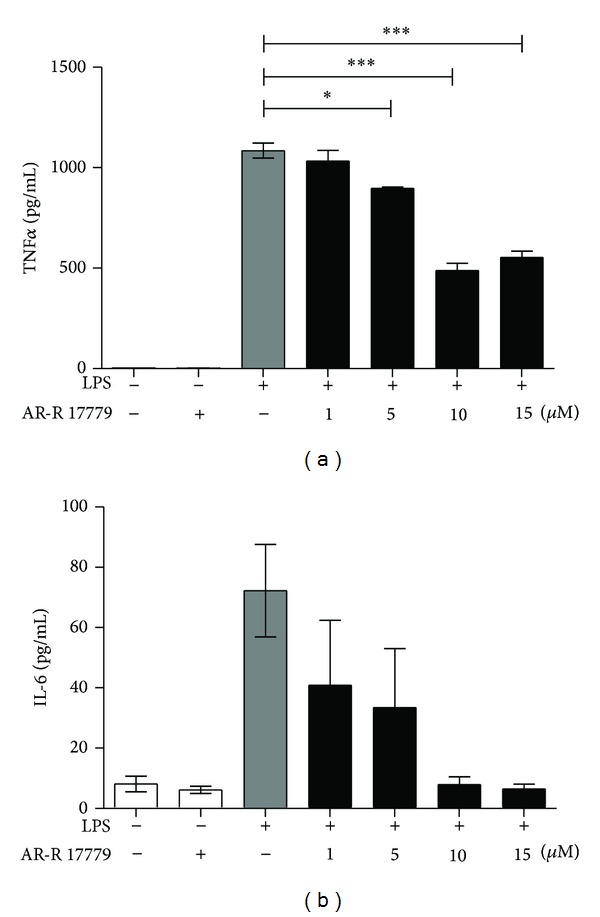
*α*7R stimulation decrease TNF*α* levels in a dose-dependent manner. Primary microglial cultures were stimulated with LPS (10 ng/mL) in combination with 1, 5, 10, or 15 µM of *α*7R agonist AR-R 17779 for 4 h and levels of TNF*α* (a) and IL-6 (b) were determined in cell culture supernatants by ELISA. 1 µM *α*7R agonist AR-R 17779 did not influence the LPS induced TNF*α* response, however, 5, 10, and 15 µM significantly decreased the TNF*α* levels (a). Treatment with LPS and *α*7R agonist AR-R 17779 did not influence the level of IL-6 between groups (b). Graph represents pooled data from 3-4 independent experiments, *n* = 3-4/group. Data are expressed as mean ± SEM, *P < 0.05, ***P < 0.001.

Previous evaluation of the anti-inflammatory role of *α*7R in microglial cultures has mostly been based on nicotine or acetylcholine (ACh) stimulations in combination with proinflammatory stimuli such as LPS [24, 25] and few studies have evaluated other *α*7R ligands. In the current study we investigate the effect of the selective *α*7R agonist AR-R 17779 in microglial cultures. After 4 hours stimulation with LPS, in combination with increasing doses of *α*7R agonist AR-R 17779, we detected a significant decrease in TNF*α* levels in the cell culture supernatant (Figure 3(a)). Further, we also investigated the effect of *α*7R agonist AR-R 17779 on the pleiotropic cytokine IL-6. Interestingly, there was a numerical dose-dependent decrease in IL-6 levels, similar to the TNF response; however, this did not reach significance (Figure 3(b)). Hence, our results support the earlier studies in microglial cultures using nicotine and ACh [24, 25] as well as in hippocampal cultures [23]; that is, *α*7R agonist have an anti-inflammatory effect on microglia.

The mechanism behind the suppressed cytokine response is intriguing. In microglia, the properties of the *α*7R differ from the neuronal *α*7R [25]. Rather than functioning as a conventional ligand-gated ion channel causing Ca^2+^ influx, the *α*7R activates intracellular pathways including phospholipase C (PLC) and release of Ca^2+^ from intracellular stores [25]. In the periphery, several different intracellular signaling pathways have been suggested to be involved in the anti-inflammatory effect mediated via the *α*7R, including the JAK2/STAT3 pathway [38, 39], MAPK [24, 40], and NF*κ*B [41]. At present, only MAPK have been demonstrated to participate in the anti-inflammatory effect after *α*7R agonist treatment in microglia [24], whether other signaling pathways also are engaged remains to be investigated.

### 3.4. *α*7 Receptor Agonist AR-R 17779 Polarize Microglia into Mox Phenotype

**Figure 4:**
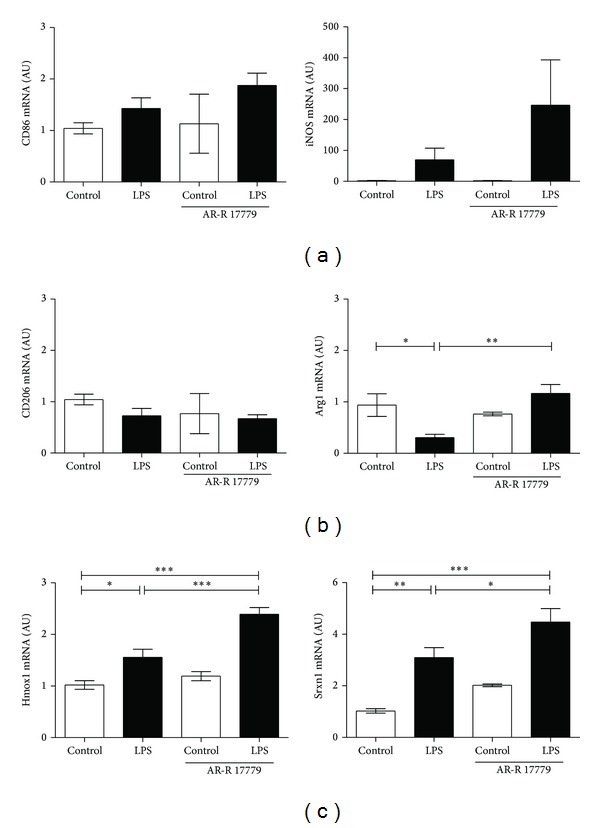
*α*7 receptor agonist AR-R 17779 polarize microglia towards Mox phenotype. Primary microglia cultures were stimulated with LPS (10 ng/mL) with or without *α*7R agonist AR-R 17779 (10 µM) for 4 h. Cells were collected and gene expression of (a) M1 markers CD86 and iNOS, (b) M2 markers CD206 and Arginase 1 (Arg1) and (c) Mox markers heme oxygenase (Hmox1) and sulfiredoxin-1 (Srxn1) was investigated. Treatment with LPS and *α*7R agonist AR-R 17779 did not influence microglial expression of M1 markers and M2 marker CD206; however, M2 marker Arg1 was downregulated by LPS, and upregulated by the combination of LPS and *α*7R agonist AR-R 17779 ((a)-(b)). Mox markers Hmox1 and Srxn1 were both upregulated by LPS and *α*7R agonist AR-R 17779 treatment (c). Graph represents pooled data from 4-5 independent experiments, *n* = 6–8/group for all except Control AR-R 17779; *n* = 2. Gene expression was normalized to YWHAZ and analyzed using ΔΔCT method. Data are expressed as mean ± SEM, *P < 0.05, **P < 0.01,***P < 0.001.

It was recently shown that in the periphery, oxidative stress drives macrophages towards a novel macrophage phenotype (Mox) mediated via activation of nuclear factor erythroid 2-like factor 2 (Nrf2) [10]. Interestingly, the *α*7R agonist PNU282987 is neuroprotective and decreases inflammation in adult brain injury, an effect mediated via Nrf2 [23]. Based on these finding we sought to determine the microglial phenotype in the current experiments. By real-time PCR we analyzed M1 markers CD86 and iNOS, M2 markers CD206 and Arginase 1 (Arg1), and Mox markers heme oxygenase (Hmox1) and sulfiredoxin-1 (Srx1) [10]. None of the M1 markers, CD86 and iNOS, or M2 marker, CD206 was regulated by LPS or the combination of LPS and *α*7R agonist AR-R 17779 (Figures 4(a)-4(b)). Interestingly, M2 marker Arg1 was downregulated by LPS and gene expression was then normalized with the combination of LPS and *α*7R agonist (Figure 4(b)). Further, Mox markers Hmox1 and Srxn1 were both significantly upregulated by LPS and AR-R 17779 treatment (Figure 4(c)), suggesting that *α*7R stimulation drives microglial cells towards the Mox phenotype. The exact role of the Mox phenotype* in vivo* remains to be investigated although Mox macrophages have been proposed to exert anti-inflammatory and anti-oxidizing effects* in vivo *[42]. Possibly, the decreased TNF*α* levels after *α*7R agonist could partly be due to the Mox phenotype.

## 4. Conclusions

In line with what is seen in patients with neurodegenerative disorders and in experimental models of adult brain injury, HI in neonatal mice decrease the expression of the *α*7R. This regulation proposes an important role for *α*7R also in the developing brain. Further, we demonstrated an anti-inflammatory effect of the *α*7R agonist AR-R 17779 on microglial cells, possibly, partly due to upregulation of the *α*7R transcript after stimulation with *α*7R agonist, but potentially also partly due to microglial polarization towards the Mox phenotype. Thus, our data suggest a role for the *α*7R in neonatal brain injury and support the anti-inflammatory role of the *α*7R in microglial cultures, suggesting that *α*7R stimulation could enhance the polarization towards a reparative Mox phenotype.
